# Highlight: From Prey to Pathogen—The Role of Predation in Driving Virulence

**DOI:** 10.1093/gbe/evae170

**Published:** 2024-08-19

**Authors:** Casey McGrath

Opportunistic pathogens are typically benign microbes that can sometimes cause infections in individuals with weakened immune systems or when they gain access to sterile areas of the body. Unlike obligate pathogens, opportunistic pathogens do not depend on host infection or transmission for survival, making it difficult to determine the factors driving the evolution of virulence in these microbes. Nevertheless, given their potential to cause severe infections in immunocompromised patients and the increasing prevalence of multidrug resistance in these species, understanding the factors that drive their virulence is crucial. While researchers have hypothesized that predation of bacteria by protists—larger eukaryotic microbes—might increase the virulence of opportunistic pathogens, there has been conflicting evidence from evolution experiments. In a new study published in *Genome Biology and Evolution*, researchers from North Carolina State University showed that the presence of the protist predator *Tetrahymena thermophila* increased the virulence of the opportunistic pathogen *Serratia marcescens* (Hopkins et al. 2024). These findings suggest that predation plays a dual role in microbial evolution, influencing both the survival strategies and pathogenic potential of bacterial prey.

The study, undertaken by research associate Heather Hopkins, graduate student Christian Lopezguerra, and postdoctoral researcher Meng-Jia Lau and led by assistant professor Kasie Raymann, involved the experimental evolution of *S. marcescens*, a widespread bacterium known to produce a red pigment that has been linked to both the pink film lining bathroom tiles and cases of “miraculous” bleeding statues and communion wafers. *S. marcescens* can also cause opportunistic infections in a wide variety of plant and animal hosts. In humans, *S. marcescens* commonly occurs in the gastrointestinal tract, where it is generally considered harmless, but it is also known to cause dangerous nosocomial infections in the respiratory and urinary tracts. “The ability of *S. marcescens* to inhabit so many diverse hosts and environments makes it a perfect model for studying bacterial adaptation and the evolution of virulence,” notes Raymann. “I started working on *S. marcescens* as a postdoc because it is an opportunistic pathogen of honey bees, but I quickly became intrigued by its broad host range and ability to thrive everywhere, including my bathroom.”

**Fig. 1.**
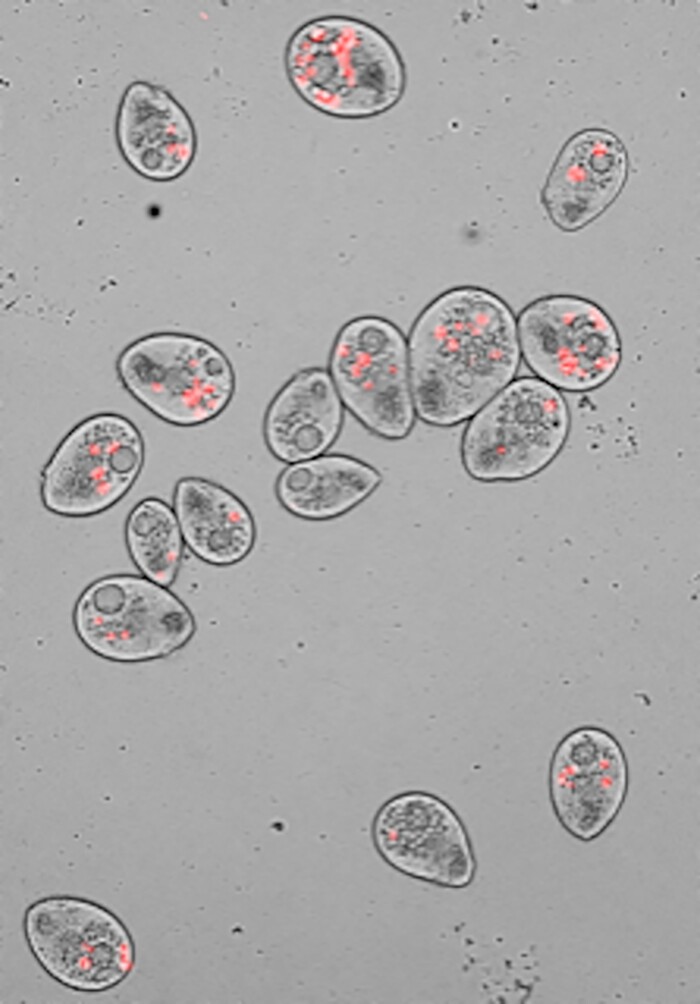
Image showing predation of fluorescently labeled *Serratia marcescens* KZ19 by *Tetrahymena thermophila.* Reproduced from Hopkins et al. (2024).

The researchers evolved *S. marcescens* KZ19, a strain known to cause opportunistic infections in honey bees, for 60 d. Six lines were grown in the presence of the ciliate *T. thermophila*, a generalist protist predator, and six lines were grown without any protists (Fig. 1). They then sequenced the *S. marcescens* lines, as well as individual isolates from each line, and characterized both the genotypic and phenotypic changes in the different populations.

Surprisingly, a large number of mutations occurred at the same site or in the same gene across multiple evolved lines. These parallel mutations occurred significantly more often than expected by chance, indicating positive selection in all evolved populations to adapt to their respective environments. “It was surprising to see the same mutations across replicates, and to be able to see it in such a small time frame was powerful,” said Hopkins. “This reflects how evolution is constantly shaping the world we live in, even if it's not obvious on a daily basis.”

Many of the parallel mutations occurred within a single protein–protein interaction network, affecting proteins in the BarA–UvrY two-component regulatory system and the three core proteins of the Rcs phosphorelay system. The BarA–UvrY system is believed to regulate the production of toxins, quorum sensing, motility, and several metabolic functions, while the Rcs system regulates motility, capsule biosynthesis, biofilm formation, and virulence. According to the study's authors, mutations in these genes are likely to have major impacts by altering the expression of multiple downstream genes.

Interestingly, there were some notable differences in the types of mutations that occurred in the lines exposed to *T. thermophila* compared to the lines evolved in the absence of a protist predator. Most strikingly, *S. marcescens* populations subject to predation exhibited several mutations in genes involved in forming biofilms, assemblages of bacteria that adhere tightly to surfaces and are enclosed in an extracellular matrix. While biofilm formation is a natural defense mechanism against predation, biofilms are also heavily associated with virulence and antimicrobial drug resistance.

To determine the effects of the observed mutations, the researchers assessed the growth, biofilm formation, predation resistance, and virulence of isolates from each evolved population. As expected, *S. marcescens* populations exposed to *T. thermophila* exhibited stronger predation resistance and biofilm formation than the predator-free populations. Remarkably, the predator-exposed lines also showed greater virulence, resulting in higher mortality of honey bees when the bees were fed the bacteria. In fact, there was a strong correlation between predation resistance and virulence in these lines, suggesting that predation can indirectly select for or maintain virulence in opportunistic pathogens.

While these results point to a complex interplay between predator–prey interactions and the evolution of virulence in opportunistic pathogens, this study is only a small piece of the puzzle according to Raymann, whose overall goal is to investigate how various environmental pressures impact the evolution of opportunistic pathogens. Investigations involving longer evolutionary experiments and different opportunistic bacteria are likely to further expand our view of the forces driving the evolution and virulence of opportunistic pathogens. Conducting such experiments is not without its challenges however. According to Raymann and Hopkins, potential obstacles include difficulties in linking specific mutations with their functional roles and accounting for the effects of abiotic and biotic factors present in the natural environment. Nevertheless, such studies are necessary to provide a better understanding of opportunistic pathogens and to help guide the development of more effective treatment strategies for combating bacterial infections.
